# Influencing factors and improvement paths of manufacturing innovation performance: Configuration analysis based on TOE framework

**DOI:** 10.1371/journal.pone.0294630

**Published:** 2023-11-20

**Authors:** Youcai Ma, Zhaobing Cui

**Affiliations:** College of Economics and Management, Shandong University of Science and Technology, Qingdao, China; Hebei Agricultural University, CHINA

## Abstract

Innovation is the first driving force to lead development, how to improve manufacturing innovation performance has become a hot topic. Based on 47 listed companies in the computer, communication and other electronic equipment manufacturing industry in the A-share market, this paper adopted the Fuzzy set qualitative comparative analysis (fsQCA) to explore the influencing factors of technology, organization and environment on the innovation performance of manufacturing industry and the improvement path. The findings are as follows: (1) A single condition is not a necessary condition for high innovation performance in manufacturing industry, but government support plays a key role in improving innovation performance in manufacturing industry. (2) There are two improvement paths for high innovation performance in manufacturing industry, which are specifically explained as “technology-environment dual improvement path” and “technology-organization-environment collaborative improvement path”. (3) The improvement of innovation performance in the manufacturing industry is the result of multiple factors, showing the characteristics of “all paths lead to the same destination”. Different manufacturing enterprises have different paths to improve innovation performance based on their actual conditions. Based on these findings, this study may provide some implications for the effective improvement of manufacturing innovation performance.

## 1.Introduction

As one of the representatives of advanced manufacturing, the computer, communication and other electronic equipment manufacturing industry plays an important role in promoting the high-quality development of China’s manufacturing industry. With the deepening of the new round of scientific and technological revolution, the electronic equipment manufacturing industry carries out technological innovation and continuously improves innovation performance, which provides impetus for the high-quality development of the economy [1]. At present, the development of the electronic equipment manufacturing industry has shown good resilience, but there are still many dilemmas in the process of implementing high-quality development. On the one hand, the lack of core technology has become an important reason for hindering the development of enterprises [2]. The manufacture of chips is still unable to meet market demand and needs to rely on foreign technology. On the other hand. Although the electronic equipment manufacturing industry is growing rapidly, it still has to face the challenge of slowing market demand, and the key to improving its international competitiveness lies in innovation. The level of innovation performance of the electronic equipment manufacturing industry indirectly reflects the innovation status of the manufacturing industry. If the level of innovation performance of the electronic equipment manufacturing industry is too low, it is not conducive to the sustainable development of the manufacturing industry. And the improvement of innovation performance is of great significance in cracking the current predicament of manufacturing industry which is big but not strong [3]. Therefore, it has become an important issue to explore how to improve manufacturing innovation performance from the perspective of the electronic equipment manufacturing industry.

Many factors that can influence corporate innovation performance have discussed in recent researches. In terms of external factors, government subsidies [4], innovation network structure characteristics [5], industrial agglomeration [6] all have an impact on firm innovation performance. In contrast, such as innovation capacity [7], corporate profitability [8], technological absorptive capacity [9], corporate social responsibility [10], more scholars have focused on the role of internal factors on innovation performance. In summary, most studies focused on the independent influence mechanism of different factors on the innovation performance of manufacturing enterprises. However, there were few studies on the configuration effects among the multiple factors. The influencing factors of manufacturing innovation performance are not independent of each other, and the path of manufacturing innovation performance improvement is a complex process of multi-factor combination and synergistic interaction.

In order to address the issues mentioned earlier, this paper focuses on the electronic equipment manufacturing industry and uses fsQCA method to explore the path to improve the innovation performance of manufacturing industry. And this study mainly tries to answer the following three questions: (1) What are the influencing factors of manufacturing innovation performance under the TOE framework? (2) What conditions exist to promote the improvement of manufacturing innovation performance? (3) What types of paths exist to improve manufacturing innovation performance?

The core innovation point of this paper is to construct a TOE theoretical analysis framework that affects the innovation performance of manufacturing industry, and identify the “technology-environment dual improvement path” and “technology-organization-environment collaborative improvement path”. From the perspective of electronic equipment manufacturing industry, it provides the evidence of manufacturing innovation performance improvement. In terms of theoretical contributions, on the one hand, this paper contributes to the research on the influence of multiple factors on manufacturing innovation performance, which is an effective supplement to the existing researches which mostly focus on a single factor. On the other hand, this paper explores the improvement path of manufacturing innovation performance and provides research basis for the high-quality development of manufacturing industry.

## 2.Theoretical model construction

### 2.1 TOE framework

The TOE framework was originally proposed by Tornatzky and Fleischer in the process of technological innovation [11]. TOE contains technological, organizational, and environmental factors [12]. The advantage of the TOE framework over other models is that it identifies internal and external influences and provides a more comprehensive research perspective [13]. In recent years, TOE framework has been used in the manufacturing industry. For example, TOE framework was used to identify the factors that influence the performance of service transformation in manufacturing firms [14]. TOE framework was used to identify the factors that influence firm’s digital innovation [15]. In addition, it examined the factors influencing innovation performance in the pharmaceutical manufacturing industry [16].

**Fig 1:**
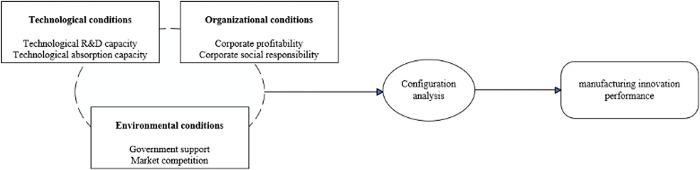
Framework of theoretical model of manufacturing innovation performance.

In summary, this paper analyses the TOE theory to classify the factors influencing manufacturing innovation performance into three aspects: technological, organizational and environmental factors. The theoretical model framework affecting manufacturing innovation performance is constructed, as shown in Fig 1.

### 2.2 Technological conditions and manufacturing innovation performance

Technological innovation is the creative activity of an enterprise to acquire new technologies, which can improve enterprises’ innovation performance [17,18]. The driving force of technological innovation depends more on internal factors, the continuous accumulation of enterprise technology will increase the level of innovation performance. Of course, it is not enough to improve the innovation performance of enterprises only through internal research and development. For some enterprises with a long R&D cycle and a high difficulty coefficient, technological introduction can achieve twice the result with half the effort. Technological progress is the driving force to improve the innovation performance of enterprises, and enterprises can promote technological progress through independent research and development or technological introduction [19]. In this paper, two secondary conditions of technological research and development capacity and technological absorption capacity are taken as the technological condition level factors.

(1) Technological R&D capacity and manufacturing innovation performance. Technological research and development must be based on high-level talent. The R&D capacity of human capital can contribute to improving labor productivity, which improves the innovation performance of firms [20]. The more human capital is invested, the more innovation output will be produced [21]. The introduction of highly skilled talents can bring tacit knowledge to enterprises and affect the technological base of enterprises, especially for the introduction of cross-border human resources, which has become an important factor to promote technological spillover [22]. Relevant studies have confirmed that technological human capital can significantly affect the innovation performance of enterprises [23]. In conclusion, a firm’s technological development can rely on its R&D personnel to improve its innovation performance. There is a correlation between the technological research and development capacity and manufacturing innovation performance.

(2) Technological absorption capacity and manufacturing innovation performance. When companies want to urgently need new products in a short period of time, they may not be able to meet the product requirements with their own R&D capacity alone. Companies can try to use their own absorptive capacity to acquire technology from outside. Technological absorption capacity enables the rapid transformation of knowledge acquired from external sources into the technology needed by firms [24,25]. With a good level of absorptive capacity, firms can optimally allocate redundant resources to improve innovation performance [26]. If an enterprise has not formed the knowledge system in its field and blindly introduces the required technology, the technological absorption capacity will not match it and the expected results of an enterprise may not be achieved. Chen (2012) found that enterprises with higher technology absorption capacity have higher organizational innovation performance [27]. Therefore, the technological absorption capacity can contribute to manufacturing innovation performance.

### 2.3 Organizational conditions and manufacturing innovation performance

The characteristics of an enterprise itself will also affect innovation performance, so it is necessary to consider the influence of the characteristics of enterprises. The development of enterprises is mainly driven by profit, and the level of profitability can directly determine the strength of R&D investment. However, it is not necessarily the case that the more profit, the more innovation investment, but also depends on whether an enterprise will take social responsibility, and the fulfilment of social responsibility will bring “hidden benefits” to enterprises, enhance the level of partners and the willingness of government support. Based on the existing research results, this paper selects two secondary conditions of corporate profitability and corporate social responsibility as organizational condition factors.

(1) Corporate profitability and manufacturing innovation performance. The profitability of enterprises is directly related to the investment in innovation. For enterprises with strong profitability, managers can control business operations more effectively [28], thus promoting the formation of high innovation performance level of enterprises. Related academic studies have shown that corporate profitability has a positive impact on green product innovation [8]. There is a relationship between corporate profitability and manufacturing innovation performance.

(2) Corporate social responsibility and manufacturing innovation performance. Corporate social responsibility as a hot topic, it has attracted global attention and academic research [29]. In particular, the relationship between corporate social responsibility and corporate innovation performance has become a topic of interest [30]. The findings on the impact of CSR on corporate innovation performance can be divided into two main categories. On the one hand, corporate social responsibility can effectively promote corporate innovation. For example, active corporate social responsibility can lead to financial support [31]and increased willingness of employees to innovate [32], which improves innovation performance. In addition, CSR can contribute positively to innovation [33,34]. On the other hand, some studies suggested a negative relationship between CSR and innovation performance [35,36]. In conclusion, there is a relationship between the corporate social responsibility and manufacturing innovation performance.

### 2.4 Environmental conditions and manufacturing innovation performance

Government and the market, as external influences on firms’ innovation performance, can affect firms’ development. The government can provide support the innovation environment of enterprises. For example, in the face of the sudden outbreak of COVID-19, the government has issued relevant preferential policies to ensure the impetus for innovation and development of enterprises. Markets can create the conditions for competition, and under fierce competition, enterprises are encouraged to carry out product updates and constantly meet market demand. Combining the above two factors, this paper takes the two secondary conditions of government support and market competition as the environmental condition level factors.

(1) Government support and manufacturing innovation performance. Government support plays a crucial role in enhancing regional innovation capabilities [37]. Various government innovation policy support can encourage innovation [38]. Government subsidies are an important tool for governments to encourage firms to innovate. Government subsidies can motivate firms to undertake R&D activities and have a positive effect on their innovation efficiency [39]. Through government subsidies, firms may be able to overcome their capital constraints. There are differences in the effects of different levels of subsidies to firms. Of course, it is not the case that the higher the level of funding, the more innovative will be the output. Small and medium-sized enterprises receive less government subsidies, but they produce better results [40]. Li et al (2021) argued that there is a non-linear relationship between government subsidies and technological innovation [41]. Therefore, there is a correlation between the government support and manufacturing innovation performance.

(2) Market competition and manufacturing innovation performance. Market competition, as an external influence on managers’ decisions, affects the innovation activities of firms [42]. Under fierce competition, the innovation efficiency of firms will increase [43]. Market competition can improve innovation performance by promoting technology maturity [44] and enhancing communication among R&D teams [45]. Related research suggested that market competition may inhibit firms from green innovation [46]. Therefore, there is a link between market competition and manufacturing innovation performance.

## 3.Methods

### 3.1 FSQCA method

Fuzzy Set Qualitative Comparative Analysis (fsQCA), developed by the social scientist Charles Ragin, is a new method of social research [47]. It uses set theory to accurately and rigorously evaluate affiliations through qualitative and quantitative comparisons [48]. In short, it is mainly used to analyze how the combination of different variables affects the dependent variable.

The main reasons for choosing fsQCA method in this paper are as follows:(1) Previous studies often focused on the influence of a certain factor on manufacturing innovation performance, but ignored the influence of different factors on manufacturing innovation performance. The fsQCA method can analyze the special problem of complex preconditions. (2) The fsQCA method can combine different factors to produce the same result, so that the research object can choose its own promotion path according to its actual situation, which is more practical significance. In summary, TOE framework analyzes the factors that affect the innovation performance of manufacturing industry, and uses fsQCA method to combine the factors, so as to find out the path to improve the innovation performance of manufacturing industry.

### 3.2 Samples and data

As a technology-intensive industry, the computer, communications and other electronic equipment manufacturing industry is characterized by high investment in technological research and development and rapid product renewal [49], and its innovation performance is crucial to the development of manufacturing industry. China’s electronic equipment manufacturing industry has developed rapidly in recent years. According to the《China Statistics Yearbook on High Technology Industry》, the operating income of the electronic equipment manufacturing industry accounted for 63.05% of the operating income of the high-tech industry in 2020. Therefore, this study selected the computer, communication and other electronic equipment manufacturing industry in 2020 as the research samples. In selecting the research samples, firstly, this paper excluded all other industries except manufacturing. Secondly, ST and ST* manufacturing listed companies were excluded. Finally, companies with incomplete index data for each measurement variable were excluded. After the above screening treatment, a total of 47 companies were identified as the research samples.

The sample data that measured corporate social responsibility originated from Hexun.com. Other corporates data were obtained from the China Stock Exchange Market and Accounting Research Database (CSMAR). Manufacturing enterprises listed on the stock exchange in 2020 were selected for research, and the data interval was from December 31, 2019 to December 31, 2020.

### 3.3 Measures

Manufacturing innovation performance. According to previous studies, patenting activity was considered to be a good proxy for corporate innovation [50]. Therefore, manufacturing innovation performance was measured by the number of patent applications filed by firms.

Technological research and development capacity. R&D activities require skilled personnel to carry on the pursuit of knowledge and maintain a competitive position [51]. In this paper, we measured technological research and development capability by using the proportion of the number of R&D personnel in the total number of employees.

Technological absorption capacity. The technological absorption capacity of enterprises depends on the diversity of its own knowledge [52]. Some scholars had shown that knowledge diversity can be represented by R&D intensity as a proxy variable [53]. R&D investment can improve a company’s technological absorption capacity by enhancing its ability to transform and develop external knowledge [25]. Therefore, technological absorption capacity was measured by R&D investment as a percentage of operating revenue [54].

Corporate profitability. It was usually measured by the ratio of net profit to total assets [8]. In this study, corporate profitability was measured by the ratio of operating income to operating expenses.

Corporate social responsibility. Based on the practice of Zhu [55], this study used data from listed companies’ social responsibility reports on Hexun.com.

Government support. Government support can stimulate the initiative of enterprises’ R&D activities and thus improve their innovation performance [56]. In this paper, we measured government support by taking the logarithm of the amount of government subsidies [4].

Market competition. This paper refers to the practice of Aghion [57], the Lerner index is used to measure the competitive position of the manufacturing industry. It is calculated as (operating revenue-operating costs-selling expenses-administrative expenses)/operating revenue. The specific measurement methods for all variables in this study are listed in Table 1.

**Table 1:** Variables and measurements.

Variables	Symbol	Description
Manufacturing innovation performance	MIP	the number of corporate patent applications
Technological research and development capacity	R&DC	Number of R&D personnel/ Total number of employees
Technological absorption capacity	TAC	R&D investment/Operating income
Corporate profitability	P	Operating income/ Operating cost
Corporate social responsibility	CSR	three dimensions: Shareholders, employees and social responsibility
Government support	GS	Log ^(the amount of government subsidy)^
Market competition	MC	(operating revenue-operating costs-selling expenses-administrative expenses)/operating revenue

## 4.Empirical analysis and results

### 4.1 Calibration of data

Calibration is the process of assigning a set membership score to a case [58]. In this paper, we use the direct calibration method to calibrate the raw data. According to the calibration criteria of Fan et al., as well as the actual situation of the case, the full membership point of one result variable and six condition variables is set to 0.95, the crossing point is set to 0.5, and the completely non-membership point is set to 0.05 [59]. By calculating the data of each variable, the calibration values of each condition and result are obtained, as shown in Table 2.

**Table 2:** Condition and result calibration.

Condition and Result		Calibrate		
		Full Affiliation	Crossover	Incomplete Affiliation
Result variable	MIP	708.600	62.000	10.400
Technological conditions	R&DC	0.585	0.215	0.099
	TAC	0.194	0.072	0.040
Organizational conditions	P	2.796	1.442	1.152
	CSR	27.070	22.080	8.341
Environmental conditions	GS	8.483	7.364	6.739
	MC	0.495	0.188	0.073

### 4.2 A One-variate analysis of necessity

Before the adequacy analysis of conditional configuration, it is necessary to test whether a single condition exists the necessary conditions for manufacturing innovation performance. Consistency level is regarded as the necessary condition for measuring manufacturing innovation performance. When consistency level is greater than 0.9, it can be considered as the necessary condition for manufacturing innovation performance. According to the test results of the fsQCA3.0 software, as shown in Table 3, the consistency of all conditions is less than 0.9. Therefore, a single condition cannot constitute a necessary condition for manufacturing innovation performance, and it is necessary to conduct configuration analysis for each condition.

**Table 3:** Necessity analysis of manufacturing innovation performance.

Condition variable	High Manufacturing Innovation Performance		Non-High Manufacturing Innovation Performance	
	Consistency	Coverage	Consistency	Coverage
R&DC	0.634	0.583	0.583	0.688
~ R&DC	0.660	0.553	0.646	0.694
TAC	0.685	0.635	0.566	0.673
~ TAC	0.647	0.537	0.693	0.739
P	0.632	0.616	0.618	0.774
~ P	0.768	0.610	0.693	0.707
CSR	0.713	0.598	0.671	0.722
~ CSR	0.668	0.612	0.626	0.737
GS	0.827	0.744	0.518	0.598
~ GS	0.553	0.472	0.779	0.853
MC	0.616	0.571	0.645	0.768
~ MC	0.750	0.622	0.640	0.681

### 4.3 Adequacy analysis of conditional configuration

Different from the necessity analysis of a single condition, the configuration analysis no longer simply considers a single condition, but analyzes the adequacy of results caused by different configurations of multiple conditions. When setting the case frequency threshold, it is necessary to decide according to the number of research samples. For small and medium-sized samples, the frequency threshold can be set to 1. On the conformance threshold, the conformance level for determining adequacy should not be lower than 0.75 [58]. According to the data characteristics in this paper, the consistency threshold is set to 0.75, and the number of samples in this paper is 47, so the frequency threshold is set to 1. To avoid contradictory configurations, the PRI consistency threshold is set to the lowest acceptable standard of 0.7. In the final analysis of conditional configuration results, the results of three solutions will appear at the same time, which are the reduced solution, the intermediate solution and the complex solution. The intermediate solution was mainly used in the analysis of the results. The reduced solution was used to judge the core and boundary conditions of different configurations. If it appears in both the intermediate solution and the reduced solution, the condition can be determined to be the core condition [60]. If it occurs only in the intermediate solution but not in the reduced solution, the condition can be judged to be an edge condition [57]. If it does not appear in the intermediate solution, it can be judged as blank. The results are shown in Table 4.

**Table 4:** Configuration analysis of high innovation performance in manufacturing industry.

Conditional configuration	Configuration 1	Configuration 2	Configuration 3	Configuration 4
R&DC	⊗	●	●	●
TAC	●	●	●	⊗
P	⊗	●	⊗	●
CSR		⊗	●	●
GS	●	●	●	●
MC	⊗			⊗
Consistency	0.943	0.949	0.982	0.967
Original Coverage	0.373	0.326	0.338	0.260
Unique Coverage	0.108	0.064	0.040	0.035
Consistency Of Solution	0.936			
Coverage Of Solution	0.567			

Note: ● or ● indicates that the condition exists, ⊗ or ⊗ indicates that the condition does not exist; ● or ⊗ denotes the core condition, and ● or ⊗ denotes the edge condition. Blank space indicates that the condition may or may not exist.

Configuration 1(~ R&DC* TAC*~ P* GS*~ MC): This configuration is a sufficient condition configuration of high innovation performance in manufacturing industry, which is composed of weak technological research and development capacity, strong technological absorption capacity, low corporate profitability, strong government support and low market competition pressure. This configuration can explain about 37.3% of the cases of high innovation performance in the manufacturing industry, and is the main path to improve innovation performance of the manufacturing industry. The lack of technological research and development capacity as a core condition means that the enterprise’s independent innovation capacity is weak, while the existence of technological absorption capacity as a core condition indicates that the introduction of foreign technology can be well applied to the enterprise innovation. Meanwhile, adequate government support will reduce the risk of insufficient funds caused by technology introduction. It can stimulate the enthusiasm of manufacturing enterprises to take the initiative to achieve technological progress and improve innovation performance in a short time, so the path is named “technology-environment dual improvement path”. Taking TCL Technology Group Corporation as an example under this path, in 2020, the strategic acquisition of semiconductor enterprises has achieved strategic reserves in the semiconductor field through the company’s strong technology absorption capacity. TCL Technology Group Corporation is the enterprise that receives the most government subsidies in the research sample, which enables it to have sufficient funds to absorb the acquired technology and improve its innovation performance. With the continuous integration of new technologies into enterprise innovation, combined with the characteristics of the enterprise’s own products, the independent innovation ability of enterprise will be enhanced, not only to make the enterprise’s products more intelligent, but also to make breakthroughs in the field of semiconductor key core technology.

Configuration 2 (R&DC* TAC* P*~ CSR* GS): This configuration is composed of strong technological research and development capacity, strong technology absorption capacity, high corporate profitability, low level of corporate social responsibility and strong government support. This configuration can explain about 32.6% of the cases of high innovation performance in the manufacturing industry. Compared with configuration 1, the technological research and development capacity and profitability of enterprises are stronger, indicating that enterprises pay more attention to independent innovation and make more profits. The increase in profits of manufacturing enterprises will enhance the willingness of enterprises to invest in innovation, and provide a financial guarantee for the improvement of enterprise innovation performance. Taking Guizhou Space Appliance Corporation Limited as an example of this path, the enterprise is one of the enterprises integrating scientific research and production, with strong independent research and development ability, many technical fields in the leading level in the country, and many advanced manufacturing technologies in the world. The manufacturing enterprise has rich product types, broad market prospects and great profit opportunities.

Configuration 3 (R&DC* TAC* ~P* CSR* GS): This configuration is composed of strong technological research and development capacity, strong technological absorption capacity, low corporate profitability, high level of corporate social responsibility and strong government support. This configuration can explain about 33.8% of the cases of high innovation performance in the manufacturing industry. If an enterprise has a strong strength in independent research and development and technology introduction, it will continuously stimulate the vitality of innovation. Of course, this may require a large amount of resource investment, and the profit of the enterprise may be relatively reduced. But it also leads to a high level of innovation performance. For example, Appotronics Corporation Limited, an exemplary company following this path, has a strong R&D team that not only has a large number of local researchers, but also recruits many overseas professionals to form an international R&D team. It has a good intellectual property management system, pays attention to the protection of intellectual property rights, and is the first patent applicant to propose the fluorescence laser technology route in China. It also focuses on social responsibility and creates a more satisfying experience for consumers with technological innovation.

Configuration 4 (R&DC* ~TAC* P* CSR* GS*~ MC): This configuration is composed of strong technological research and development capacity, weak technological absorption capacity, high corporate profitability, high level of corporate social responsibility, strong government support and low market competition pressure of manufacturing high innovation performance sufficient conditions. This configuration can explain about 26.0% of the cases of high innovation performance in manufacturing. Compared with configuration 3, although enterprises have weak technology absorption capacity, they have strong profitability and can have enough resources to invest in innovation. At the same time, they have a strong sense of social responsibility and pay attention to corporate honor, which is also conducive to the improvement of enterprise innovation performance. Take Anker Innovations Technology Corporation Limited, a sample company under this path, as an example. The enterprise actively adopts effective management methods to prevent commercial corruption, requires employees to be honest and self-disciplined, constantly increases customer satisfaction through innovation, and has a high reputation, which plays an important role in attracting talents and increasing employee loyalty, so that employees can spontaneously participate in enterprise innovation.

Configuration 2, Configuration 3 and Configuration 4 all show that high innovation performance of manufacturing industry is generated under the joint action of technology, organization and environment, so they are named “technology-organization-environment collaborative improvement path”.

### 4.4 Robustness test

In order to make the results more convincing, the robustness of each configuration needs to be tested. Therefore, this paper attempts to increase the PRI consistency threshold from 0.7 to 0.75 for testing, and the test results are shown in Table 5. The test results show that the configuration results before and after adjusting the PRI consistency threshold are basically consistent, indicating that the research results in this paper are relatively robust.

**Table 5:** Robustness test results.

Conditional configuration	Configuration 1	Configuration 2	Configuration 3	Configuration 4
R&DC	⊗	●	●	●
TAC	●	●	●	⊗
P	⊗	●	⊗	●
CSR		⊗	●	●
GS	●	●	●	●
MC	⊗	●		⊗
Consistency	0.943	0.950	0.982	0.967
Original Coverage	0.373	0.292	0.338	0.260
Unique Coverage	0.116	0.045	0.043	0.035
Consistency Of Solution	0.937			
Coverage Of Solution	0.548			

## 5.Discussion

### 5.1 Technology-environment dual improvement path

**Fig 2:**
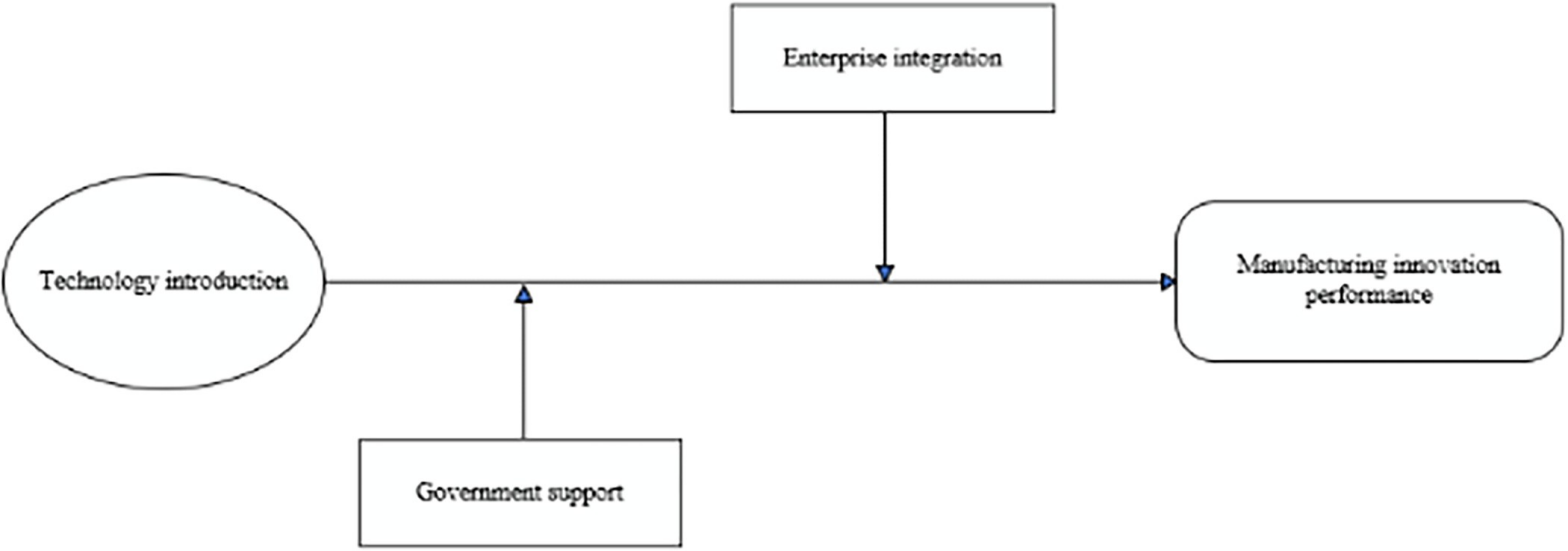
Technology-environment dual process to improve manufacturing innovation performance.

There is an effective configuration (configuration 1) under the “technology-environment dual improvement path”. The characteristic of this configuration is that it can make full use of external resources and integrate them into enterprise innovation. In other words, when the independent innovation ability is insufficient and the enterprise’s own research and development status is not optimistic enough, it can make full use of government support to introduce advanced technology, so as to improve innovation performance. The introduction of advanced technology can indeed improve the innovation performance of enterprises [61]. Of course, manufacturing enterprises must have the ability to digest and absorb foreign technology, otherwise, it is difficult to convert imported technology into production capacity [62]. Therefore, under this path, configuration 1 can be regarded as the “technology integration support type”. Fig 2 describes the process of the “technology-environment dual improvement path” to improve manufacturing innovation performance.

### 5.2 Technology-organization-environment collaborative improvement path

There are three effective configurations (configuration 2, 3 and 4) in the “technology-organization-environment collaborative improvement path”. These three configurations have the three conditions of technology, organization and environment at the same time, and government support and technological research and development capacity play a key role in the process of improving the innovation performance of the manufacturing industry, which also fully shows that the development of enterprises cannot be separated from the guidance of the government, enterprises need to grasp the current development environment, constantly enhance the ability of independent innovation, and deploy the relationship between technology, organization and environment.

Configuration 2 shows a high level of profitability and strong technological absorption capacity. Enterprises can have sufficient funds to invest in technology research and development to improve their independent innovation ability, and can also introduce technology to better improve their innovation performance by using their own learning and integration ability. This configuration fully shows that the size of corporate profits can directly determine the amount of innovation investment. The investment of R&D funds affects the innovation activities of enterprises [63]. Moreover, R&D investment has a positive impact on innovation output [64]. Therefore, this configuration can be regarded as “cash driven”.

Compared with configuration 1, configuration 3 has two core conditions: technological research and development capacity and corporate social responsibility, which further proves the rationality of configuration 3. If the enterprise can take the initiative to assume social responsibility, it will be easier to obtain the recognition and innovation knowledge of stakeholders [65]. This configuration fully explains the difference with configuration 2. In contrast, although the profits earned by the enterprise are small, based on the sense of corporate social responsibility and the development of an enterprise depends on technological innovation, the enterprise will still be encouraged to actively invest in innovation research, and the government will also provide more support to the enterprise. Therefore, this configuration can be viewed as “responsibility-technology integration driven”.

**Fig 3:**
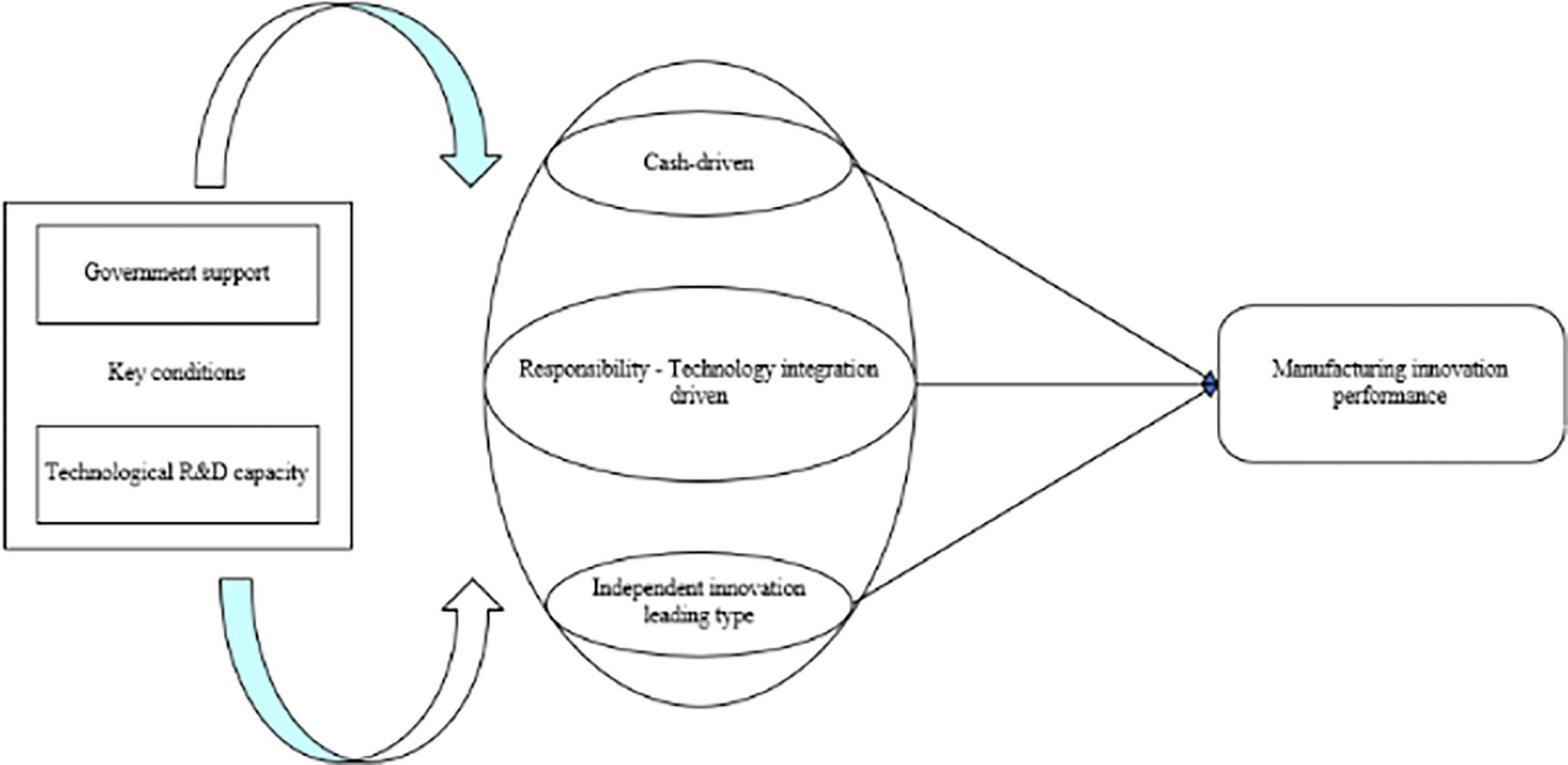
Technology-organization-environment collaborative improvement of manufacturing innovation performance process.

Configuration 4 also shows a high level of corporate social responsibility, and also has strong profitability. Different from Configuration 1, enterprises mainly rely on independent research and development to improve their own innovation performance and pay more attention to social responsibility. When enterprises establish a good image, they will attract more consumers and increase corporate profits. This configuration fully demonstrates that independent innovation can effectively improve innovation performance without relying on external technology. For the enterprise itself, independent innovation is of great significance for mastering core technologies and is the main source of improving innovation performance in manufacturing industry [66]. Therefore, this configuration is regarded as the “independent innovation leading type”. Fig 3 shows the process of “technology-organization-environment collaborative improvement path” to improve manufacturing innovation performance.

To sum up, if an enterprise really wants to improve the quality of innovation, it cannot be achieved only by unilateral factors. It requires a combination of different conditions, and there are interactive effects between different conditions. Different enterprises have certain differences in resources and environment, and their own conditions are good or bad. It can make up for the defects caused by some conditions and achieve the purpose of improving the innovation performance of manufacturing enterprises. Government support exists in all four configurations, so manufacturing enterprises should make full use of government guidance. The innovation activity of electronic equipment manufacturing industry has high risk, which may lead to insufficient innovation ability of enterprises. This is why there is government support in all four configurations, which can provide a guarantee for enterprise innovation activities [67]. The government can support enterprises to innovate through tax incentives, research and development subsidies, government procurement and other policies [68]. For market competition, the presence or absence of market competition in the four configurations does not mean that market competition is not important. The selected sample cases are in the unified manufacturing industry, which indicates that the competitiveness of each enterprise is relatively different, but enterprises can enhance their competitiveness by improving innovation performance.

## 6.Conclusion and enlightenment

### 6.1 Conclusions

This paper constructs TOE configuration analysis framework for manufacturing innovation performance, and uses the fsQCA method to explore the configuration effects of technological, organizational and environmental factors on manufacturing innovation performance and the improvement path. The results are as follows:

(1) From the perspective of individual conditions, technological research and development capacity, technological absorption capacity, corporate profitability, corporate social responsibility, government support and market competition cannot be the necessary conditions for improving manufacturing innovation performance alone. From the perspective of the four configurations, government support plays a key role, and market competition pressure has little impact on manufacturing innovation performance.

(2) There are two improvement paths for high innovation performance in manufacturing industry. The first improvement path is the “technology-environment dual improvement path”, which is explained as the dual enhancement of technological and environmental factors. The second improvement path is the “technology-organization-environment collaborative improvement path

”, which is explained as the synergistic improvement of technology, organization and environment. According to the configuration of each condition, under the dual improvement path, it can be defined as “technology integration support type”. Under the path of collaborative improvement, with government support and technological research and development capacity as the key conditions, it is mainly divided into “cash driven”, “responsibility-technology integration driven” and “independent innovation leading type”.

(3) The improvement of manufacturing innovation performance is the result of the joint action of different factors. Different manufacturing enterprises will develop innovation performance improvement paths suitable for enterprise innovation development according to their own conditions.

### 6.2 Enlightenment

The conclusions of this study provide the following suggestions for manufacturing enterprises to improve innovation performance.

(1) Manufacturing enterprises should grasp the relevant support of the government, make good use of the resources and preferential policies given by the government, and truly integrate them into enterprise innovation to improve their own innovation performance.

(2) Manufacturing enterprises should enhance the ability of independent innovation. Although the introduction of technology can improve the innovation performance of enterprises in a short period of time, it is difficult for enterprises to obtain core technology from the external environment, and ultimately rely on independent research and development. The enhancement of independent innovation ability of enterprises can overcome technical difficulties, and can bring a qualitative leap in enterprise innovation.

(3) All manufacturing enterprises should strengthen the synergy between technological, organizational and environmental factors, and enterprises should effectively and reasonably formulate relevant policies to improve their innovation performance according to their own conditions.

### 6.3 Limitations and prospects

There are still some shortcomings in the research process of this paper: Firstly, this paper takes the electronic equipment manufacturing industry as research samples, and the results of other industries may be different. The measurement method of manufacturing innovation performance can be improved, and the influence of different manufacturing industries on innovation performance can be removed, so that more manufacturing enterprises can be included in the research samples to make the results more accurate. Secondly, future studies can introduce more conditional variables into the TOE framework, such as organizational learning capacity and management governance capacity, so as to more comprehensively explain the path to improve manufacturing innovation performance.

## Supporting information

S1 Data (CSV)

S2 Data (CSV)
